# Examining patient choice and provider competition under the National Health Insurance Fund outpatient cover in Kenya: does it enhance access and quality of care?

**DOI:** 10.1186/s12913-024-12021-9

**Published:** 2024-12-18

**Authors:** Jacob Kazungu, Edwine Barasa, Matthew Quaife, Justice Nonvignon

**Affiliations:** 1 Health Economics Research Unit, KEMRI Wellcome Trust Research Programme, Nairobi, Kenya; 2 Center for Tropical Medicine and Global Health, Nuffield Department of Medicine, University of Oxford, Oxford, UK; 3 Department of Epidemiology and Population Health, London School of Hygiene and Tropical Medicine, London , UK; 4 Department of Health Policy, Planning and Management, School of Public Health, University of Ghana, Legon, Accra Ghana; 5 Health Economics and Financing Programme, Africa Centres for Disease Control and Prevention, Addis Ababa, Ethiopia

**Keywords:** Patient choice, Provider competition, Access, Quality, National Health Insurance Fund, NHIF, Kenya

## Abstract

**Background:**

While patient choice and provider competition are predicted to influence provider behaviour for enhancing access and quality of care, evidence on provider perceptions and response to patient choice and provider competition is largely missing in low-resource settings such as Kenya. We examined provider and purchaser perceptions about whether patient choice and provider competition influenced provider behaviour and enhanced access and quality of outpatient care in Kenya.

**Methods:**

We conducted a qualitative study to explore this across two purposefully selected counties. We conducted 15 in-depth interviews (IDIs) with health facility managers and National Health Insurance Fund (NHIF) staff across the two counties. We examined these across five areas summarised as either local market conditions or patient feedback following the Vengberg framework.

**Results:**

NHIF members’ choice of outpatient facilities compelled private and faith-based providers to compete for members while public providers did not view choice as a way of spurring competition. Besides, all providers did not receive any information regarding the exit of NHIF members from their facilities. Providers felt that that information would be crucial for their planning, especially in enhancing service accessibility and quality of care. Most providers ensured the availability of drugs, provided a wider range of services and leveraged on marketing to attract and retain NHIF members. Finally, providers highlighted their redesign of service delivery to meet NHIF members’ needs whilst enhancing the quality-of-care aspects such as waiting time and having qualified health workers.

**Conclusion:**

There is a need for NHIF to share NHIF members’ exit information with providers to support their service delivery arrangements in response to NHIF members’ needs. Besides, this study contributes evidence on patient choice and provider competition and their influence on access and quality of care from a low-resource setting country which is crucial as NHIF transitioned to the Social Health Authority.

## Introduction

Patient or population choice of providers and competition among healthcare providers is increasingly gaining recognition for potentially enhancing the access and quality of care across many health systems [1–4]. While healthcare markets often deviate from the classical economic model prediction where demand and supply define price formation, the healthcare sector operates ‘quasi-markets’ where prices are often fixed and regulated by a purchaser such as a Ministry of Health or a social health insurance organisation [1, 5]. In such settings, instead of providers competing on price, they compete for patients [2].

Proponents of patient choice have argued that allowing patients to choose providers enhances patients’ freedom, strengthens the doctor-patient relationship, and places the patient at the centre of care to allow them to make the best judgements about the quality of care they need [6]. In line with the standard economic theory, it is assumed that patients are rational decision-makers and take into account all important factors prior to choosing a health facility [7]. Similarly, providers are predicted to respond to patients’ choices by adjusting their service delivery accessibility and quality of care to both attract and retain patients within their facilities [2].

While there are several studies on patient choice and provider competition, most of these studies have focussed on high-income countries [1–4, 8, 9]. Their evidence is however mixed where some indicated patient choice and provider competition enhanced access and quality whereas some did not show evidence of enhanced access and quality of care. In the United Kingdom for instance, Dixon et al. showed that patient choice and provider competition resulted in positive responses from providers leading to enhancing better access to services and the quality of care delivered [2]. Besides, evidence from another study in Stockholm by Wohlin et al. showed that allowing patient choice through the introduction of competition for hip and knee replacements resulted in the reduction of waiting times, leading to the improvement of access to care and also enhanced the quality of care [4]. On the other hand, in a study across 13 Swedish Primary Care centres, Vengberg et al. did not find any evidence of enhanced clinical quality but it was indicated that patient choice and provider competition enhanced provider awareness of patients’ service accessibility concerns which incentivised providers to improve [3]. A quantitative study conducted in Ghana, a lower-middle-income country, showed that provider competition enhanced access and the quality of care for hypertension patients [10].

Even though the evidence on whether provider competition enhances access and quality of care is mixed, there is a consensus on the need to understand the context or circumstances in which patient choice and provider competition occur [11]. For instance, Goddard et al. postulated that it is more valuable to consider the circumstances in which competition may work well to achieve desired health system goals rather than whether it is “good” or “bad” [11]. Further, Barros et al. argue that provider competition could be leveraged as an instrument to achieving health systems goals rather than a goal on its own and having patient choice may not necessarily result in provider competition or vice versa [12]. Consequently, understanding the circumstances and ideals for provider competition is crucial for countries designing policies that encourage patient choice especially in low- and middle-income countries such as Kenya.

In Kenya, the National Health Insurance Fund (NHIF) is the main public purchaser with key roles in the establishment of service entitlements for the beneficiaries, selection and contracting of providers and provider payment [13, 14]. Over the years, NHIF has undergone several reforms aimed at transforming it into a strategic purchaser of healthcare services by primarily expanding the benefits package, introducing new premium rates and introducing new provider payment mechanisms [15]. In 2015, the NHIF introduced a reform aiming at extending the service coverage by incorporating an outpatient cover before which, the NHIF provided an inpatient cover only [16]. Critical to the introduction of outpatient cover was that members were required to choose an outpatient provider before accessing care with an option to change the provider once every quarter (three months) while providers were to be paid using capitation [17]. In line with existing literature, making members choose and change facilities was anticipated to (1) drive up provider competition where providers would strive to attract and retain NHIF members for higher capitation amounts and (2) incentivize providers to enhance access to services and the quality of care provided [3, 4].

Consequently, the introduction of outpatient cover under NHIF in 2015 in Kenya provides an opportunity for critically examining patient choice and if or whether that drives provider competition in a low-resource country such as Kenya and further understand the extent to which patient choice and provider competition influence access and quality of care across NHIF-contracted outpatient facilities. While patient choice and provider competition are expected to influence provider behaviour for enhancing access and quality of care, evidence on provider perceptions and response to patient choice and provider competition is largely missing in low-resource settings such as Kenya. In this study, we examined provider and purchaser perceptions of patient choice and provider competition, particularly whether these meet the ideals for influencing provider behaviour to enhance access and quality of outpatient care in Kenya. This evidence is crucial as NHIF transitioned to the Social Health Authority (SHA) in October 2024 although SHA members will still have to select providers to access outpatient services and SHA will contract and pay providers.

## Methods

### Study setting

We conducted a qualitative study in two purposefully selected counties in Kenya: Nyeri and Makueni, following an approach described in another paper [18]. The counties were included in this study to represent a county which had piloted the national-level universal health coverage (UHC) programme (Nyeri) and a county (Makueni) that had both not implemented the national-level UHC pilot but also had a locally run UHC programme. The use of the UHC programme implementation criteria in the inclusion of the study counties was to provide useful information to support the UHC scale-up in Kenya, particularly through NHIF which has been selected as the ‘vehicle’ to drive the UHC agenda in Kenya.

Kenya has a pluralistic health system characterised by having both public and private healthcare providers in equal share [19]. Kenya runs a devolved government comprised of a national government and 47 semi-autonomous counties [20]. Health is one of the devolved functions where counties are responsible for service delivery and management of primary and secondary facilities while the national government is responsible for policy design and management of tertiary facilities [20, 21]. Overall, health facilities in Kenya are categorised into four tiers comprising six levels of care [22]. Tier 1 comprises community health services categorised as Level 1 of care. Tier 2 comprises the primary healthcare services which include the Dispensaries and Clinics (Level 2) and Health Centres (Level 3). Tier 3 comprises the sub-county and County referral hospitals categorised as Level 4 and 5 hospitals respectively. Tier 4 is made of the National referral facilities categorised as Level 6 or tertiary hospitals in Kenya.

### Conceptual framework

To examine how patient choice and provider competition influenced access to healthcare services and the quality of care under the NHIF’s outpatient cover, we developed a conceptual framework based on previous work by Vengberg et al. [3]. Our framework postulates that while patient choice directly influences competition among healthcare providers, the competition among providers may be used by patients to inform their decisions to choose or exit a given provider. However, for patient choice and provider competition to incentivize providers to enhance access and quality of care, certain circumstances need to be met including (1) providers need to have the intention to compete within the market; (2) There need to be sufficient providers to enhance the competitiveness of the market; (3) providers need to be made aware of patient choices i.e., providers need to be notified when a patient chooses or exits from their facility; (4) providers need to have/plan measures to both attract and retain patients and (5) providers need to be able to analyse information on patients’ preferences. Consequently, Vengberg et al. summarise these into two main factors: (1) the local market conditions, and (2) feedback from patient choice [3].

Local market conditions relate to the first three conditions (1, 2 and 3) highlighted above whereas feedback from patients relates to the last two conditions above (4 and 5). It is postulated that in the action of the provider competition, providers would respond to the patient’s needs and particularly ensure that patients obtain their needed services (access to care) and that the quality of the services they get is high enough in a bid to both attract and retain them.

### Study design and data collection

**Fig. 1:**
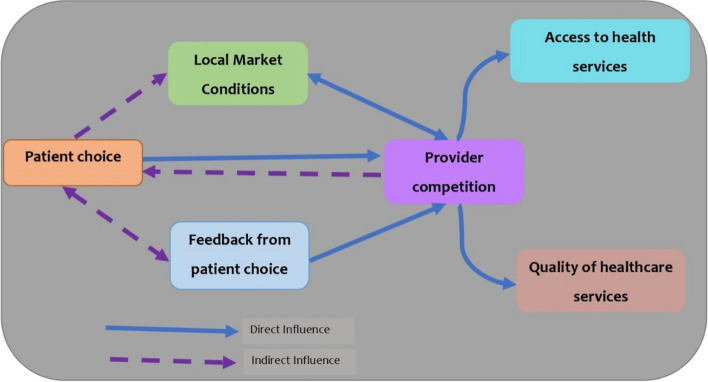
Conceptual framework

We conducted a qualitative cross-sectional study where we interviewed NHIF managers and health facility managers across the two purposefully selected counties. Data were first collected from NHIF managers from each of the two selected counties. These managers were purposefully included in the study as they were either heading the NHIF branch office in the selected county or were mandated with roles related to NHIF member registration and/or contracting of healthcare providers. Second, we randomly selected one facility from each of the levels 2 to 4 of care in Kenya including the three ownership types: private-for-profit, faith-based, and public providers. Table 1 summarises the number and levels of care included in the data collection. We conducted in-depth interviews (IDIs) with both NHIF managers and health facility managers, each taking 30 to 90 min. The interviews were conducted in English and audio recorded. We developed an IDI guide based on the conceptual framework (Fig. 1) adapted from [3].

**Table 1:** Summary of the number of in-depth interviews conducted per county

IDI stakeholder	Makueni county	Nyeri county	Total
County NHIF managers	2	1	3
Public hospital	2	2	4
Private-for-profit facilities	2	2	4
Faith-based facilities	2	2	4
**Sub-total IDIs**	**8**	**7**	**15**

### Data analysis

We transcribed audio records verbatim in English. Data were analysed following a framework analysis approach following four key steps: reading and re-reading transcripts to familiarise with key themes aligned with the framework, identifying repeated patterns, and coding data according to the framework [23]. The effect of patient choice and provider competition on access were assessed following the availability and affordability dimensions of access as proposed by Penchansky and Thomas [24]. Data management and coding were conducted using Nvivo 12 [25].

## Results

### Local market conditions

#### Providers’ intention to compete in the market

Only respondents across private and faith-based facilities indicated the need to compete for NHIF members whereas public providers did not see the need for competition because either way NHIF members would select their facilities as they were mostly the only public facilities in their areas or have an extensive range of services. For these public facilities, respondents had no intention or motivation to increase their capitated NHIF member lists because they didn’t have a ‘money-minded’ motivation, unlike, for instance, the private sector.

*“We don’t* [compete for NHIF members]. *I wouldn’t say that we compete for NHIF members because most of our clients are unique. One*,* we attract the ones that are in the low-income levels… second*,* whenever we offer services*,* we don’t limit the number of services that we offer. Third*,* we don’t look at the money aspect*,* but you look at the service aspect.”* IDI 026 Public facility Nyeri County.

*“The competition is very stiff. I would say the competition is very stiff. So we try our best to give the best services and we try to provide all the services within our facility. Not sending the clients out there* [to other facilities].*”* IDI 028 Private facility Nyeri County.

#### Availability of sufficient providers to permit competition

All providers indicated the availability of other healthcare providers within a close range (as close as within a kilometre) and experienced some level of competition for NHIF members, especially for private and faith-based facilities.

*“We have one* [other health facility] *just next here* [in this building], *a few in town*,* we have a big hospital in town*,* and then also there are clinics in town. There’s* [another one] *like ten kilometres from this place.”* IDI 026 Public facility Nyeri County.

*“*[There are other health providers around]…*Like some meters away. Some like 30 meters from here*,* there is a private hospital. I think a kilometre from here there is another private hospital*,* a public hospital*,* I think. They are just nearby.”* IDI 028 Private facility Nyeri County.

While a range of providers existed, some providers especially public providers and faith-based felt that there was not much competition for NHIF members between them and other providers. This was associated with the range of services they provided in the regions and also the fact that they were perceived to be largely cheaper than private facilities.

While some private providers viewed competition as a threat to their survival given their dependence on the capitation they received from NHIF, there was a general consensus that competition was a positive thing. Generally, competition among providers was thought of as a way to spur innovation among providers to always do better than the competitor.

*“I think the competition has a positive end. Because we try to look for innovative ideas. What is the competitor doing that we’re not doing? What are they delivering that we’re not? So it also keeps us on our toes so it’s a positive thing.”* IDI 028 Private facility Nyeri County.

#### Providers’ awareness of patient choice

While providers indicated they were getting information on the list of NHIF members who had chosen their facility at the beginning of every quarter, they indicated they did not receive any information regarding the exit of NHIF members from their facilities. Providers felt that that information would be crucial for their planning, especially in enhancing service accessibility and quality of care which were perceived as the major reasons (apart from NHIF members relocating) for NHIF members’ change of facilities.

*“No*,* we don’t* [get any information on patient exit from NHIF]. *We only get the list of*,* the active members at the start of the quarter.”* IDI 030 Faith-based Nyeri County.

*“I think so…the information will be very useful because every patient that exits it means*,* he or she’s not satisfied*,* with a certain service*,* so I think it’ll help us improve. If the service is not available*,* we may bring it back. If there’s a drug that is not available*,* we also buy it. Yeah. It’ll help*,* the feedback is good.”* IDI 030 Faith-based Nyeri County.

### Patient feedback

#### Providers analyse NHIF members’ information

Overall, NHIF did not provide health providers with information on the reasons for NHIF members’ exit from facilities. While members were required to fill out a form when changing a facility, the data was not availed to providers. However, some providers reported having functional mechanisms within their facilities that they leveraged to understand NHIF members’ reasons for choosing or exiting their facilities. These facilities utilised suggestion boxes and did periodic patient exit surveys to collate this information. Besides, private and faith-based facilities monitored the number of capitated NHIF members every quarter and could follow up with some members especially where there were many exits.

*“The NHIF does not give us any information about the reasons why members leave our facilities but we monitor our numbers every quarter to see if there has been any big differences or reduction….* [if there are] *we follow up and sometimes we try to call those who have left to know why.”* IDI 002 Private facility Makueni County.

Overall, while providers felt NHIF members could leave their facility due to the quality of care they provided, largely, they felt that members would leave because they had relocated, were no longer active NHIF members or for other reasons. They, however, expressed a need to receive information about the reasons for NHIF member exits from their facilities so that they can, where necessary, enhance their service delivery to align with members’ needs.

*“There are small sometimes large changes in the number of NHIF members capitated here but NHIF does not tell us why for example members decide to leave our facility for another facility… I think this information would be very useful to us so that we know if is it drugs or specialists we don’t have or what areas we need to improve for them to be satisfied.”* IDI 043 Faith-based Makueni County.

#### Plans to attract and retain NHIF members

On providers’ plans to attract and retain NHIF members within their facilities, health providers highlighted several strategies that they were either executing or planning to execute to attract more NHIF members whilst retaining those that had already selected their facilities.

##### Marketing of private and faith-based facilities

Private and faith-based providers leveraged on marketing to attract more NHIF members. These facilities engaged in activities such as outreach programs in both urban and rural settings to market their services through medical camps. Some faith-based facilities indicated engaging community members on weekly health awareness programs through a local radio station where they would also share information on the range of services they offered and NHIF-related information including asking residents to choose their facility.

*“Our hospital is also vigilant on medical camps. Whenever we hold those, we try to promote healthcare in the interiors and to people who cannot reach the hospital, we always have a customer care desk where NHIF inquiries [are taken up and we] advise them on choosing facilities during the camps….also there is a local radio station that is available, for most of the patients who can access the hospital. We [use it to] give pro-health talks every Tuesdays, we always end with NHIF matters whereby we advise the client, encourage them to enrol and of course advertise our hospital after the health talk.”* IDI 029 Faith-based facility Nyeri County.

##### Ensuring the availability of drugs

All health providers interviewed indicated the need to ensure the availability of drugs as a way to both attract and retain NHIF members. To ensure the availability of drugs, healthcare providers indicated the use of strategies such as sourcing medications from different suppliers, especially for private and faith-based facilities that had more flexibility in determining who to source from, when and negotiating for costs of the supplies unlike across public providers.

*“So those drugs are available here compared to other facilities, like public hospitals. Yes, we have members who have selected public hospitals, then they come back to us because in the public, there’re no drugs.”* IDI 030 Faith-based facility Nyeri County.

*“So the issue of affordability, that is something that is always our challenge because there’s a lot of competition but through Meds, we’re able to get benefits because we buy [commodities and drugs] in bulk. So we enjoy that benefit of the price going down because of that kind of bargaining power we have.” *IDI 043 Faith-based Makueni County.

Besides, some providers, especially private and faith-based highlighted the use of drug compounding at the facility level to ensure that required drugs were always available in required doses when the drug is not readily available in the market.

*“Sometimes the consultant requires some drug which requires unique doses, and they’re not commercially available. So we do make sure that [we make them available]. Like now, neonates, which are being born might require very unique syrup, which is not available as an injection. We do that kind of preparation within the hospital so that we can cover that gap.”* IDI 029 Faith-based facility Nyeri County.

Additionally, all facilities highlighted the use of the list of NHIF drugs required and ensured their availability within the facility at the beginning of the quarter.

*“NHIF has a list [of drugs] that has been given to us that states what should be covered. So we ensure that we have all of them.”* IDI 003 Public facility Makueni County.

##### Providing a wider range of services and having the required health workforce

All facilities highlighted that they were currently providing a wider range of health services needed by NHIF members and had the right set of health workforce, especially specialists to attend to the needs of NHIF members. Other facilities especially private and faith-based indicated they were in the process of expanding their range of services including constructing new and larger outpatient wings and hiring more staff.

*“Being a faith-based institution, we have a large scope of services from which the patients benefit from the NHIF services. That includes our radiology department, our maternity, and our dental unit. There are also our ENT and our surgical packages. So we advise them that if they choose [this facility], they’re able to benefit from most of these services.”* IDI 029 Faith-based facility Nyeri County.

*“We do the best we can, of course, they know we have qualified personnel. We have the best physicians in town, we have medical officers, we have nurses, and they also know we offer a wide variety of services like X-ray, and ultrasound, so these people know when they come here, and our lab is well equipped. They know when they come here, they’ll get almost all the services they need, I think also that one plays a role. They also see the ambulance they know they can be transported if there is an issue.”* IDI 002 Private facility Makueni County.

On the other hand, respondents from public facilities did not need to market their facilities but leveraged the fact that they were the only public facility in their areas. However, they supplemented this by providing a wider range of services that were often unrestricted to for instance the number of visits an NHIF member could go for outpatient care.

*“The reason they choose us is because we are the only government facility within here. Also, … we are not giving them conditions on the amount of services that they’re entitled to. Other institutions which are private or mission would limit the services that people get.”* IDI 026 Public facility Nyeri County.

##### Having quality products such as medicines

Providers also attracted and retained NHIF members by providing high-quality services and products such as medicines. Providers recognised that commodities were essential to NHIF members and they needed to provide the best quality so that the members could get healed sooner to resume their duties. Approaches such as abiding by required standards of care, NHIF recommended list of commodities and international recommendations aided them in providing high-quality commodities. Besides, some facilities leveraged on their quality control departments/units to guarantee all supplied commodities are of high quality and for instance not expired.

*“We have a very nice quality control laboratory. So we ensure that whatever comes to our hospital must have gone through that process in addition to what the Pharmacy and Poisons Board recommends for our products in this country.”* IDI 029 Faith-based facility Nyeri County.

##### Enhancing user experiences

Enhancing user experiences and satisfaction was one of the strategies employed by healthcare providers to both attract and retain NHIF members at their facilities. To enhance user experiences, facilities strived to make care processes more efficient in order to reduce waiting times and system downtimes. Some private and faith-based facilities also invested in building larger outpatient wings to accommodate more patients and provide a wider range of services. Some facilities also collected feedback from clients and acted on the feedback they got to enhance user experience. Besides, some of the facilities reported were either already using or planning to introduce a health information management system that would fast-track the management of patient information and patient flow at the facility thereby reducing the time patients spent at the facility and the overall speed and efficiency in the delivery of care.

*“The way we deliver those services and customer relations and customer satisfaction, we make sure those customers will come back.”* IDI 031 Private facility Nyeri County.

*“Currently we are expanding, there’s a new building that is coming up and the building is mainly an outpatient block. So the building will mainly prioritise outpatient services… which will be more spacious. We will have more consultation rooms.”* IDI 030 Faith-based facility Nyeri County.

##### Building and fostering trust

Health providers indicated that they strive to provide the right care whilst talking to patients with respect which helps build trust. All providers strongly emphasised the need to build trust with NHIF members. To build trust, providers leveraged on providing the recommended care, adequate engagement of patients through the care process, being responsive to patients’ needs by acting on NHIF members’ feedback and being respectful when interacting with NHIF members. Then patients become their tool of marketing as they spread through word of mouth to their friends and family members who also end up choosing their facilities.

*“Building good relationships and trust with NHIF members [is also key]…. For example, if you come here and you have been treated well, then you feel like you should be coming to this hospital, so through experience, now you want to be a member of this hospital. [Then, by] word of mouth, friends, relatives who have been treated there, they refer them here, they tell them to go to [facility X] it’s a good place, yeah.”* IDI 003 Public facility Makueni County.

*“Also I think being a mission [faith-based] hospital, gives us an advantage… Cause the perception out there… there’s trust in a mission hospital compared to private.”* IDI 030 Faith-based facility Nyeri County.

### Effect of patient choice and provider competition

#### Quality of care

Allowing patients to choose and change their preferred NHIF-contracted outpatient providers was perceived by providers to directly and positively influence the quality of care provided at the facility. Quality of care was interpreted in several dimensions including the waiting time at the facility, having recommended medications (as per standard guidelines), having qualified health workers, and cleanliness of the facility.

*“Actually*,* I think this has an effect on both the access and quality of the care we are providing. Because we not only make sure the services needed are available but we also make sure that for example if it’s drugs*,* they meet the required standards*,* members don’t stay long in the queue and we have the best-trained health workers.”* IDI 002 Private facility Makueni County.

*“I think competition is an advantage because the more you have competitors*,* the more you try to increase the quality of your services. So we take competition as an advantage to us so that we can increase and continue offering better services to our clients.”* IDI 028 Private facility Nyeri County.

#### Access to care

Letting members choose and change facilities was associated with creating competition for the members among providers and hence providers strived to ensure the availability of all needed services within their facilities to prevent NHIF members from exiting.

*“We are trying to make sure that this facility is a one-stop-shop*,* whereby when the client is coming*,* it’s a hundred per cent sure that they will get the services they need because we don’t want them to go maybe to the facility which is next door.”* IDI 018 Private facility Makueni County.

Besides the availability, providers also strived to make services affordable. Affordability was achieved when healthcare providers negotiated prices of commodities with suppliers which ensured getting the commodities at a much lower price and therefore reselling these at more affordable rates which aligned with the reported lower capitation rates.

*“We know that the capitation rate we receive per person is very low but we try to ensure we have all required commodities by negotiating with suppliers to get a rate that will not make us get into losses.”* IDI 002 Private facility Makueni County.

## Discussion

While patient choice and provider competition have been hailed to positively influence provider behaviour for enhancing access and quality of care, the attainment of these depends on meeting the five ideal supplier-side conditions [2, 3]. Examining the alignment of providers to the supplier-side conditions is critical for reforms that will utilise patient choice to spur provider competition and most importantly elicit required provider behaviour for attaining health system goals. This study provides a detailed qualitative assessment of these from two purposefully selected counties in Kenya. First, we found that, unlike public providers, faith-based and private providers had intentions to compete for NHIF members even though all providers were surrounded by NHIF-contracted competitor providers. This finding is consistent with that reported in other studies [2, 3, 10]. The finding that public providers had little if any intention to compete for NHIF members is worrying and could reflect the lack of provider motivation to compete due to a lack of adequate autonomy to use funds generated at their facilities. For instance, the Public Finance Management Act requirement for facilities to remit funds to the consolidated county revenue fund (CRF) prior to use may have disincentivised public providers to compete [26]. However, this was likely not to be a major contributor as public facilities across both counties received funds directly to their facility accounts and could use the funds without remitting them to CRF. On the other hand, it is not surprising that private and faith-based providers had intentions to compete for NHIF members essentially for them to survive economically. Capitation has been highlighted as a major source of consistent income for the providers [27] and it would be expected that businesses with a profit maximisation incentive would compete for survival. Besides, these providers viewed the presence of other NHIF-contracted outpatient providers within their vicinity as threats which could have further incentivised them to intend to compete for NHIF members [2, 3].

Second, the finding that providers were not made aware of NHIF members’ reasons for exit from their facilities and therefore could not adequately analyse NHIF members’ choice information was not expected. Ideally, providers need to understand NHIF members’ reasons for changing providers for them to act on supply-side demands expressed by NHIF members. Unlike in the Swedish study [3], providers in Kenya showed interest in getting information from NHIF on NHIF members’ reasons for exits so that they can redesign their service delivery to meet the needs and expectations of NHIF members.

Third, the use of several strategies to attract and retain NHIF members at their facilities such as ensuring the availability of drugs, marketing their facilities, providing a wider range of services, and enhancing user experience as well as their service delivery changes to enhance access and quality of care are in line with predictions of competition in healthcare markets [2, 12]. Because capitation rates per individual capitated in each facility were standardized across all facilities irrespective of ownership, health providers were predicted to compete for NHIF members. Providers have been shown to enhance some aspects of health system goals such as access and quality of care in a bid to attract and retain patients [1–3, 10]. Our findings corroborate these findings. For instance, Dzampe et al. reported an increase in the quality of care as doctor density increased in Ghana [10].

We acknowledge that findings from this study may not be generalisable across the country due to the qualitative approach used. While this paper provides important nuances, there is a need for future studies to quantitatively examine whether provider choice enhances provider competition and their effect on access and quality of care. One such approach could be the use of administrative data as described elsewhere by Dzampe et al. [10] or other approaches as described by Brekke et al. [1].

## Conclusion

There is a need for NHIF to share NHIF member exit information with providers to aid their effective service delivery of care in response to NHIF members’ needs. This is particularly important as NHIF transitioned to the SHA in October 2024. Besides, there is a need to remove financial autonomy barriers among public health providers to incentivise them to compete for NHIF members which would potentially improve their efficiency, responsiveness, and their quality of care. One way is for counties to adopt the Facility Improvement Fund (FIF) which guarantees autonomy with accountability arrangements [28]. Finally, this study contributes crucial evidence on patient choice and provider competition and their influence on access and quality of care from a low-resource setting country that is crucial to advance the UHC agenda.

## Data Availability

Data and materials are available upon reasonable request to the authors (kjacob@kemri-wellcome.org).

## Abbreviations

IDIs: In-depth Interviews
MOH: Ministry of Health
NHIF: National Health Insurance Fund
UHC: Universal Health Coverage
